# The Role of Machine Learning in the Detection of Cardiac Fibrosis in Electrocardiograms: Scoping Review

**DOI:** 10.2196/60697

**Published:** 2024-12-30

**Authors:** Julia Handra, Hannah James, Ashery Mbilinyi, Ashley Moller-Hansen, Callum O'Riley, Jason Andrade, Marc Deyell, Cameron Hague, Nathaniel Hawkins, Kendall Ho, Ricky Hu, Jonathon Leipsic, Roger Tam

**Affiliations:** 1 Faculty of Medicine University of British Columbia Vancouver, BC Canada; 2 School of Biomedical Engineering University of British Columbia Vancouver, BC Canada; 3 Division of Cardiology Faculty of Medicine University of British Columbia Vancouver, BC Canada; 4 Department of Radiology Faculty of Medicine University of British Columbia Vancouver, BC Canada; 5 Department of Emergency Medicine Faculty of Medicine University of British Columbia Vancouver, BC Canada

**Keywords:** machine learning, cardiac fibrosis, electrocardiogram, ECG, detection, ML, cardiovascular disease, review

## Abstract

**Background:**

Cardiovascular disease remains the leading cause of mortality worldwide. Cardiac fibrosis impacts the underlying pathophysiology of many cardiovascular diseases by altering structural integrity and impairing electrical conduction. Identifying cardiac fibrosis is essential for the prognosis and management of cardiovascular disease; however, current diagnostic methods face challenges due to invasiveness, cost, and inaccessibility. Electrocardiograms (ECGs) are widely available and cost-effective for monitoring cardiac electrical activity. While ECG-based methods for inferring fibrosis exist, they are not commonly used due to accuracy limitations and the need for cardiac expertise. However, the ECG shows promise as a target for machine learning (ML) applications in fibrosis detection.

**Objective:**

This study aims to synthesize and critically evaluate the current state of ECG-based ML approaches for cardiac fibrosis detection.

**Methods:**

We conducted a scoping review of research in ECG-based ML applications to identify cardiac fibrosis. Comprehensive searches were performed in PubMed, IEEE Xplore, Scopus, Web of Science, and DBLP databases, including publications up to October 2024. Studies were included if they applied ML techniques to detect cardiac fibrosis using ECG or vectorcardiogram data and provided sufficient methodological details and outcome metrics. Two reviewers independently assessed eligibility and extracted data on the ML models used, their performance metrics, study designs, and limitations.

**Results:**

We identified 11 studies evaluating ML approaches for detecting cardiac fibrosis using ECG data. These studies used various ML techniques, including classical (8/11, 73%), ensemble (3/11, 27%), and deep learning models (4/11, 36%). Support vector machines were the most used classical model (6/11, 55%), with the best-performing models of each study achieving accuracies of 77% to 93%. Among deep learning approaches, convolutional neural networks showed promising results, with one study reporting an area under the receiver operating characteristic curve (AUC) of 0.89 when combined with clinical features. Notably, a large-scale convolutional neural network study (n=14,052) achieved an AUC of 0.84 for detecting cardiac fibrosis, outperforming cardiologists (AUC 0.63-0.66). However, many studies had limited sample sizes and lacked external validation, potentially impacting the generalizability of the findings. Variability in reporting methods may affect the reproducibility and applicability of these ML-based approaches.

**Conclusions:**

ML-augmented ECG analysis shows promise for accessible and cost-effective detection of cardiac fibrosis. However, there are common limitations with respect to study design and insufficient external validation, raising concerns about the generalizability and clinical applicability of the findings. Inconsistencies in methodologies and incomplete reporting further impede cross-study comparisons. Future work may benefit from using prospective study designs, larger and more clinically and demographically diverse datasets, advanced ML models, and rigorous external validation. Addressing these challenges could pave the way for the clinical implementation of ML-based ECG detection of cardiac fibrosis to improve patient outcomes and health care resource allocation.

## Introduction

### Background

Cardiovascular disease continues to be a significant global health burden, leading to 19.1 million deaths in 2022, making it the leading cause of mortality worldwide [1]. Globally, mortality from cardiovascular diseases has steadily risen from 1990 to 2021, with a 75% increase in deaths from ischemic heart disease and a 47% increase in death from stroke [1]. Cardiac fibrosis, also known as cardiac or myocardial scar, forms the underlying pathophysiological basis of numerous cardiovascular diseases. Fibrotic tissue impairs electrical conduction throughout the heart, including sinoatrial node signal generation, downstream electrical conduction, and muscular contraction leading to arrhythmogenicity, impaired cardiac output, and ultimately systemic disease [2-5].

Identification of cardiac fibrosis is an important prognostic factor, yet its identification remains a challenge despite various existing diagnostic methods due to resource limitations and testing constraints. The gold standard for cardiac fibrosis detection is endocardial biopsy which provides high specificity. However, endocardial biopsies are invasive, resource-intensive, prone to sampling bias, and risk further cardiac injury with adverse outcomes [6]. An alternative approach is to perform echocardiography, which, while noninvasive and accessible, has limitations in specificity and sensitivity [7]. Contrast-enhanced computed tomography is more readily available but evidence is preliminary for application in the identification of cardiac fibrosis [8]. The current widely used noninvasive imaging modality is cardiac magnetic resonance (CMR) with delayed gadolinium enhancement, also referred to as late gadolinium enhancement (LGE). While LGE-CMR offers high spatial resolution for scar characterization, it is cost-intensive and difficult to access [7]. To date, LGE-CMR has been most commonly used for facilitating the identification and quantification of ventricular fibrosis, although its use for atrial fibrosis is becoming more commonplace [9].

Considering the limitations of existing methods, there is a need for novel, noninvasive, low-cost, and highly accessible techniques for the detection, quantification, and characterization of cardiac fibrosis. One such identified avenue is electrocardiograms (ECGs), a widely available, inexpensive, and noninvasive technology used to document the electrical activity of the heart using a set of superficial electrodes [10]. Given ongoing human and imaging-resource constraints in medical settings, there has been a focus on noninvasive methods to streamline diagnosis and treatment. Electrophysiological data from ECGs can be used to infer structural and cardiac abnormalities, as with hypertrophy or ischemia. ECGs provide a particular advantage for the identification of cardiac fibrosis due to their noninvasiveness and accessibility in a constrained environment. Various ECG features have historically been used to identify cardiac fibrosis, with fragmented QRS (fQRS) and the calculation of a Selvester score being the most studied approaches [11].

The fQRS is traditionally assessed by identifying specific ECG features across adjacent leads, often linked to uncoordinated conduction through scarred myocardium [12]. While fQRS shows independent prognostic value for adverse cardiac events, heart failure, and mortality and has been proposed as a tool for assessing intervention eligibility, its clinical utility is limited [13-17]. Meta-analyses have reported a pooled sensitivity of 68.4% and specificity of 80.5% for detecting cardiac fibrosis; however, performance varied across populations and pathologies [18]. The fQRS is also evident in patients without fibrosis, showing low negative predictive value and poor sensitivity in some conditions, such as coronary artery disease [19-21]. It remains a nonspecific marker unable to precisely localize fibrosis [22]. Emerging techniques, such as QRS microfragmentation analysis through advanced signal processing, aim to improve the diagnostic accuracy of fQRS [23]. However, fQRS has not yet proven to be independently reliable for definitive scar identification.

Another manual method, the Selvester score, is a quantitative method for estimating left ventricular fibrosis using a standard 12-lead ECG. First introduced in 1972, it was validated through anatomical analysis and has since evolved to refine its criteria and adjust for confounding ECG factors [24,25]. Each point on the Selvester score represents a specific percentage of the left ventricular mass affected by fibrosis [26]. Studies comparing the Selvester score to LGE-CMR showed a moderate diagnostic performance, with an area under the receiver operating curve (AUC) of 0.66 and a QRS score-to-imaging Spearman correlation of 0.42 [27]. Sensitivity and specificity varied with different score thresholds; a score ≥1 had a sensitivity of 98.3% but low specificity (16.7%), while a score ≥5 showed moderate sensitivity (67.2%) and specificity (50%) [27]. The Selvester score also demonstrated prognostic value, with an association between higher scores and mortality (hazard ratio 1.16; *P*=.01) [27]. However, the score tends to overestimate fibrosis, particularly in individuals with conduction abnormalities and may lack prognostic value in some populations [28,29]. Its utility varies based on the population studied, and further research is necessary to assess its clinical applicability across different diagnostic groups.

While both methods, the fQRS complexes and the Selvester score, show potential for detecting cardiac fibrosis from ECG graphs, they are limited by the need for manual interpretation by clinicians, making them susceptible to diagnostic errors. Efforts to automate these processes, such as the algorithm by Bono et al [30] for Selvester scoring, achieved high accuracy (94%) compared to manual methods. However, both manual and automated approaches still face challenges. ECGs have become a popular target for computational analysis, particularly in the realm of artificial intelligence (AI) due to the increasing availability of data. Within AI, machine learning (ML) methods have become a dominant force for data-driven approaches. ML models leverage massive computational power to analyze large ECG datasets and can identify novel patterns that are difficult to discern by traditional human-derived methods.

Applications of ML to ECG analysis are expanding in their scope including electrophysiology for classification of cardiac abnormalities, risk stratification, prognostication, and therapeutic guidance [31]. Model evaluations for classifying cardiac electro-pathophysiologic changes from ECG have produced sensitive and specific models for cardiac contractile dysfunction, electrolyte disturbances, hypertrophic cardiomyopathy, and arrhythmias [32-34]. For example, a deep learning model for the identification of left ventricular dysfunction obtained sensitivity, specificity, and accuracy of 93.0%, 86.3%, and 85.7%, respectively. Interestingly, when this model incorrectly identified dysfunction, these individuals were more likely to develop left ventricular dysfunction over the study follow-up. This indicates the potential of ML models to identify subclinical diseases or for screening. However, research focused specifically on cardiac fibrosis detection using ML is limited, and further exploration is needed to assess the clinical utility of these tools for fibrosis localization and quantification. Expanding on recent studies and methodologies that incorporate ML for fibrosis detection will provide a more comprehensive understanding of its potential in clinical practice.

### Objective

Despite notable advancements in ML within cardiac electrophysiologic analysis, there remains a substantial gap in the application of focused ML techniques. This gap limits the full potential of ML-based methods to enhance diagnostic precision, optimize resource allocation, and improve patient outcomes. Therefore, a thorough review of current ML applications in ECG analysis for cardiac fibrosis is imperative to consolidate existing knowledge, identify effective strategies, and guide future research toward clinically viable solutions that can facilitate prompt diagnosis and better resource prioritization.

## Methods

This research focuses on the application of ML to cardiac fibrosis detection from ECGs. To capture the breadth of literature, we conducted a systematic search aligned with PRISMA (Preferred Reporting Items for Systematic Reviews and Meta-Analyses; Multimedia Appendix 1).

### Eligibility Criteria

Given the variability in publishing standards in engineering, computer science, and medicine, we considered both journal publications and conference papers. Preliminary conference abstracts with insufficient methodological detail or no corresponding papers were not included. Inclusion criteria were studies that (1) applied ML methods to predict the presence, magnitude, or location of cardiac fibrosis; (2) used ECG or vectorcardiogram (VCG) input data; (3) included information on model development, validation, and outcome metrics; and (4) were published in English. Studies were excluded if (1) ML applications did not include identification of cardiac fibrosis detection; (2) there was insufficient methodological detail or were not yet peer-reviewed (eg*,* conference abstracts, letters, case reports, or preprints); and (3) there was a primary reliance on imaging or non-ECG diagnostics for fibrosis identification. When multiple publications were reported on the same study, these were considered collectively, and all relevant results were reported together.

### Information Sources

Systematic search strategies were conducted in PubMed, IEEE Xplore, Scopus, Web of Science, and DBLP computer science bibliography to ensure all major biomedical journals and ML journals and conferences were searched. To ensure all relevant literature was identified in this relatively new research area, authors were invited to share additional studies meeting the criteria. The search was completed in October 2024.

### Search Strategy

Key terms were identified through a preliminary review of the literature and discussions with authors (JH, AM, AM-H, and RT). Each search consisted of 3 elements: cardiac electrodiagnostic methods (eg*,* “electrocardiogram”), cardiac fibrosis (eg, “myocardial fibrosis”), or known ECG identification methods of fibrosis (eg, “Selvester score”), and ML methods (eg, “deep learning”). The strategy was adjusted to the constraints of the respective database. Details of the search strategy used for each database are available (Multimedia Appendix 2). The search was conducted in October 2024. All search results published before October 2024 were included.

### Selection Process

For all stages of review management, the Cochrane review management software Covidence was used [35]. With respect to the review of literature pertaining to the use of ECG-ML for the detection of cardiac fibrosis, the identified literature underwent abstract and full-text review to determine eligibility for data extraction by 3 reviewers (JH, HJ, and CO), and data extraction was conducted by 3 reviewers (JH, AM, and HJ). Conflicts were resolved in a discussion between the authors.

### Data Extraction

Data were tabulated from each included study by one author and then verified by the second author (JH, HJ, and AM). Data from multiple publications reporting the same study were summarized in a single row.

### Data Items

Data were collected on study design, model design, and outcomes. Study design data included clinical population type, data sources, total sample size, sample size with confirmed scar, and modality used to confirm cardiac fibrosis. Data on model design included input data type, ML models used, best-performing model, validation strategy, and feature selection. Finally, outcomes included model sensitivity, specificity, positive predictive value, negative predictive value, accuracy, and the AUC.

### Data Synthesis

These data were presented in a tabulated form and a synthesis that grouped the studies by model type, including classical ML models, ensemble models, and deep learning models.

### Ethical Considerations

This review relies exclusively on publicly available information that is legally accessible to the public. Accordingly, no ethics approval was required to conduct this research per the Panel on Research Ethics of the government of Canada [36]. We further established that all included studies had ethics approval or acknowledgment of ethics waiver.

## Results

### Overview

**Figure 1:**
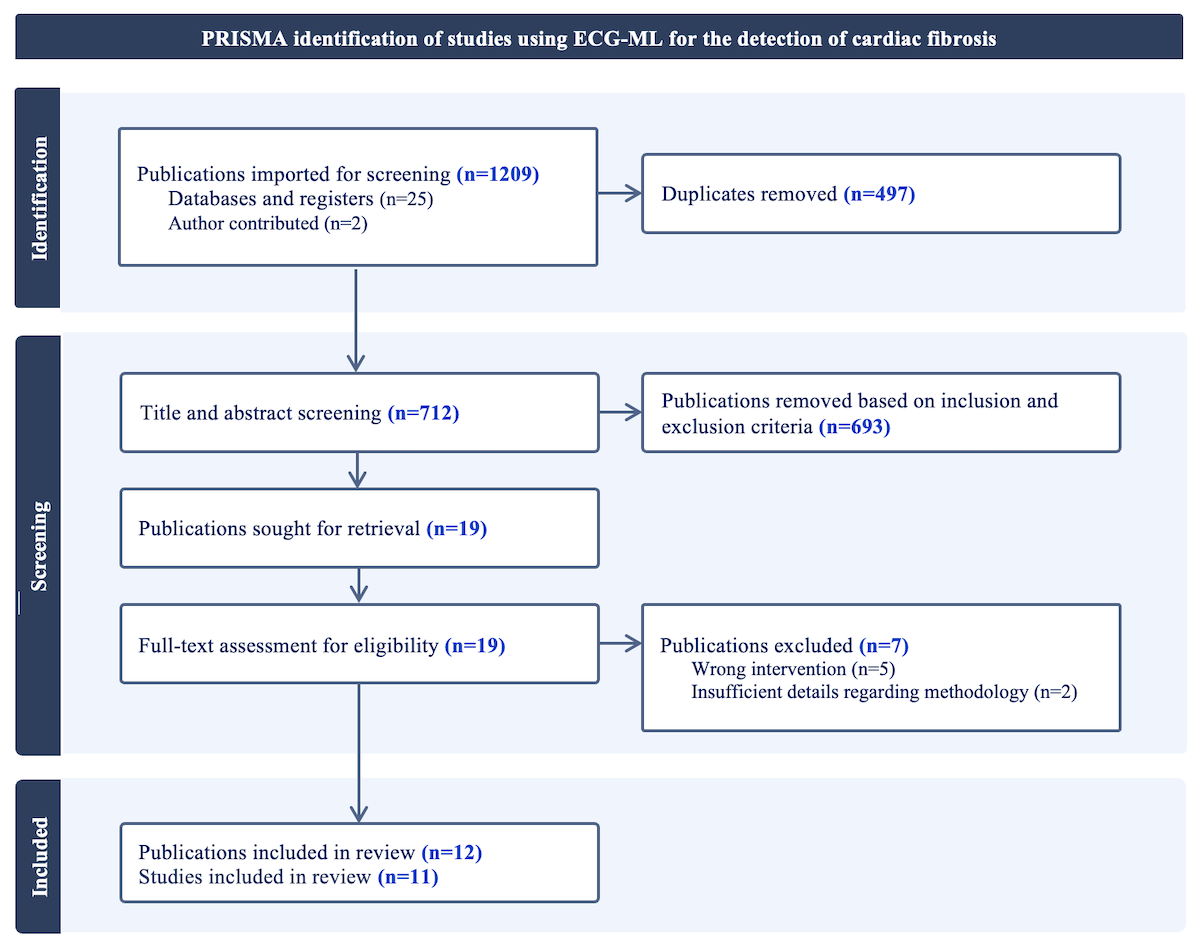
PRISMA (Preferred Reporting Items for Systematic Reviews and Meta-Analyses) 2020 [49] diagram of the identification, screening, and inclusion for the review of electrocardiogram machine learning (ECG-ML) use in the detection of cardiac fibrosis.

**Figure 2:**
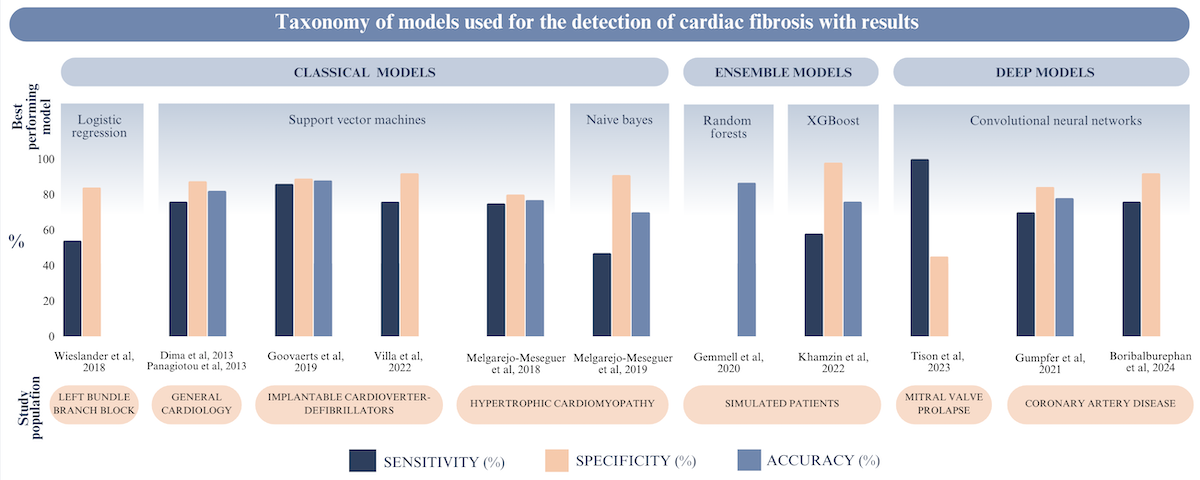
Taxonomy of machine learning models used for the detection of myocardial fibrosis across various study populations, categorized into classical, ensemble, and deep learning models. The figure presents the sensitivity, specificity, and accuracy percentages for the best-performing models.

A review of the identified literature revealed that the application of ECG-ML for the detection of cardiac fibrosis has been limited, with only 12 publications representing 11 studies to date [37-48]. Full screening data are available in the PRISMA diagram (Figure 1) [49]. An overview of features and outcomes of these studies is summarized in Tables 1 and 2 and visualized in Figure 2 [37-48]. Within ML, models are algorithms or mathematical representations that learn patterns and relationships within data to make predictions or decisions based on new, unseen data. Of the identified studies, all 11 (100%) studies used supervised learning, an approach in an ML model is trained to map input observations (ECG tracings) to the corresponding “labeled” outputs (CMR identified scar or fQRS). In this sense, the ML model learns patterns that may exist in training data to predict outcomes in a “supervised” fashion. The trained model is then used to classify (or predict) new never-before-seen test inputs from a left-out testing dataset.

**Table 1:** Summary of methods of studies that use machine learning to identify cardiac scars from electrocardiogram (ECG) data. Within the study design, N refers to the number of ECGs or vectorcardiograms included in the study, and n scar refers to the number of cases with confirmed scarring using a method other than ECG. The number of sources of study population, data sources, is included. Scar modality refers to the method used to confirm cardiac scarring. Classical models include logistic regression (LR), support vector machine (SVM), k-nearest neighbor (KNN), naive Bayes (NB), and decision tree (DT). Ensemble models include TreeBagger, random forest (RF), and Extreme Gradient Boosting (XGBoost). Deep models include convolutional neural network (CNN) and multilayer perceptron (MLP).

Study	Study design							Model design			
	Population	Data sources	N	Scar, n (%)	Scar modality	Included demographics	All models used		Best model	Development and validation strategy	Features, n
Dima et al [37], 2013	General cardiology	3	260	158 (60.7)	LGE-CMR^a^	None	SVM^b^		SVM	10-fold CV^c^	25
Wieslander et al [47], 2018	LBBB^d^	5	325	142 (43.7)	LGE-CMR	Sex and age	LR		LR	—^e^	44
Melgarejo-Meseguer et al [39], 2018	HCM^f^	1	43	25 (58)	LGE-CMR	None	SVM		SVM	5-fold CV	5
Melgarejo-Meseguer et al [48], 2019	HCM	4	80 (fQRS^g^); 300 (CMR)	42 (fQRS; 53); 130 (CMR; 43.3)	Simulated fQRS, fQRS, or LGE-CMR	Sex and age	SVM, KNN, MLP, and DT		SVM (for fQRS); NB (for fibrosis)	Bootstrap resampling (B=100)	23
Goovaerts et al [40], 2019	ICD^h^	1	616	—	ECG (fQRS)	None	SVM, KNN, NB, and TreeBagger		SVM	10-fold CV	10
Gemmell et al [41], 2020	Cardiac simulations	1	42	42 (100)	Computational simulation model	None	RF		RF	5-fold CV	—
Gumpfer et al [42], 2021	CAD^i^	1	114	—	LGE-CMR	Sex and age	CNN		CNN	6-fold CV	—
Villa et al [43], 2022	ICD	2	1932	—	ECG (fQRS)	Sex and age	SVM		SVM	10-fold CV	10
Khamzin [45], 2022	10 LBBB patients for 20,000 simulations	1	20,000	10,000 (50)	Computational simulation model	None	LR, NB, SVM, RF, XGBoost		XGBoost	5-fold CV	15
Tison et al [44], 2023	MVP^j^	1	87	21 (24)	LGE-CMR	Sex, age, and ethnicity	CNN		CNN	90:10 split	—
Boribalburephan et al [46], 2024	CAD	1	13,707	3809 (27.7)	LGE-CMR	Sex and age	Multitask CNN (ResNet)		Multitask CNN (ResNet)	5-fold CV	—

^a^LGE-CMR: late gadolinium enhancement cardiac magnetic resonance imaging.

^b^SVM: support vector machine.

^c^CV: cross validation.

^d^LBBB: left bundle branch block.

^e^Not reported.

^f^HCM: hypertrophic cardiomyopathy.

^g^fQRS: fragmented QRS.

^h^ICD: implantable cardioverter-defibrillator.

^i^CAD: coronary artery disease.

^j^MVP: mitral valve prolapse.

**Table 2:** Summary of outcomes of studies that use machine learning to identify cardiac scar from electrocardiogram data. The outcomes reported are those of the most successful model from each study. The outcomes are first reported for validation sets.

Study	Outcomes					
	Sensitivity (%)	Specificity (%)	PPV^a^	NPV^b^	Accuracy	AUC^c^

Dima et al [37], 2013	87.3	91.2	—^d^	—	89.2	—
Panagiotou et al [38], 2013	76.0	87.5	—	—	82.1	—
Wieslander et al [47], 2018	54	84	—	—	—	0.72
Melgarejo-Meseguer et al [39], 2018	75.0	80.0	85.7	66.7	76.9	—
Melgarejo-Meseguer et al [48], 2019	94 (for fQRS^e^); 47.4 (for fibrosis)	99 (for fQRS); 90.5 (for fibrosis)	98 (for fQRS); 82.1 (for fibrosis)	93 (for fQRS); 65.5 (for fibrosis)	93 (for fQRS); 70.1 (for fibrosis)	—
Goovaerts et al [40], 2019	86.0	89.0	—	—	88.0	0.94
Gemmell et al [41], 2020	—	—	—	—	76.7-86.7	0.38-0.97
Gumpfer et al [42], 2021	70.0	84.3	84.2	78.2	78.0	0.89
Villa et al [43], 2022	76.0	92.0	86.0	—	—	0.93
Khamzin [45], 2022	58.0	95	—	—	76.0	0.83
Tison et al [44], 2023	100.0	45.1	—	—	—	0.75
Boribalburephan et al [46], 2024	59.9	91.2	—	—	83.1	0.84

^a^PPV: positive predictive value.

^b^NPV: negative predictive value.

^c^AUC: area under the receiver operating characteristic curve.

^d^Not reported.

^e^fQRS: fragmented QRS.

**Figure 3:**
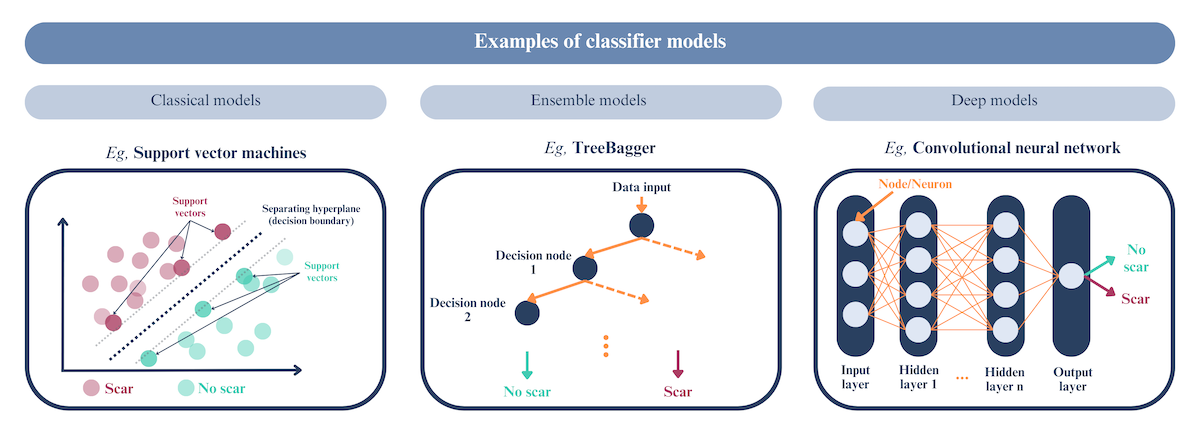
Simplified visual representation of classifier models used in machine learning for cardiac fibrosis detection from electrocardiograms (ECGs). Support vector machines encompass a family of algorithms that identify an optimal hyperplane segregating different classes of data, thereby defining a decision boundary amid data points [50]. Ensemble models, such as TreeBagger, perform classification by creating a collection of multiple bagged tree models, reducing the overfitting often seen with individual decision trees through model averaging. Deep learning, of which convolutional neural networks (CNNs) are a subclass, mimics human cognitive processing with a layer-based organization, with each layer housing nodes or neurons that allocate weight to input data and subsequently relay output data to successive nodes within the network to define a decision boundary. Unlike classical models, CNNs possess the ability to extract important features without human guidance (ie, automated feature extraction) to identify predictive patterns even with complex datasets, such as ECG data [51].

**Figure 4:**
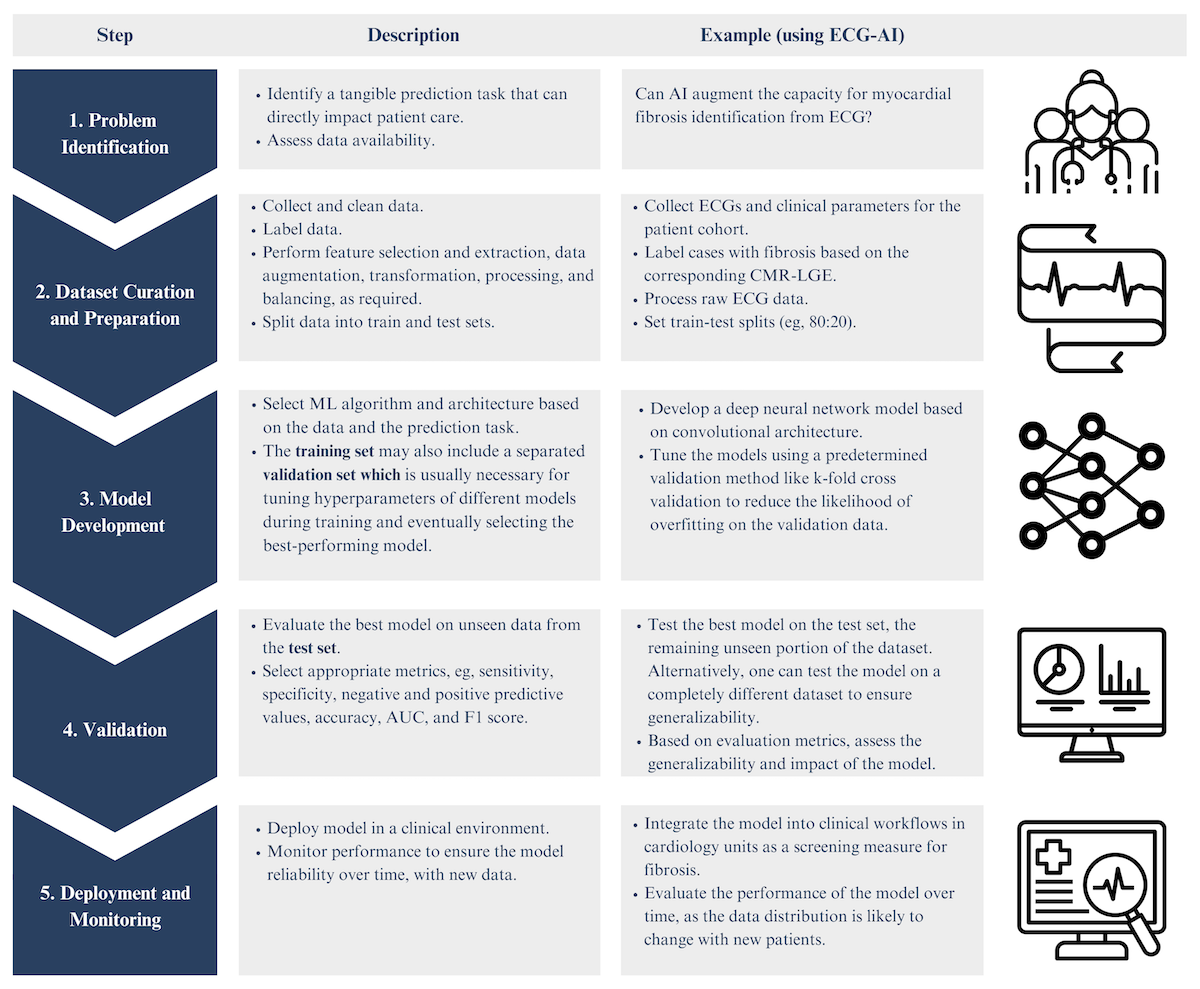
Summary of major steps in the development and implementation of a machine learning (ML) model based on Chen et al [52], with electrocardiogram (ECG) data as a case example. AI: artificial intelligence; AUC: area under the receiver operating characteristic curve; LGE-CMR: late gadolinium enhancement cardiac magnetic resonance.

Supervised learning lends itself well to diagnostic applications, where data can be classified as pathological or benign. In this case, the training data would be hand-labeled as pathological or benign by a third party, often a physician, establishing the ground truth. This ground truth serves as the definitive reference against which the ML model’s predictions are compared to evaluate accuracy. Models trained under the supervised learning approach can be further subclassified into classical models, ensemble models, and deep learning models, and are reported in these subcategories below. Visual representations of examples from each subtype can be found in Figure 3 [50,51]. Some studies applied a single model, while others compared several types of models. The translational application of ML in health care unfolds through various stages, including problem identification, dataset curation and preparation, model development (ie*,* model training and tuning), model validation, and deployment and monitoring, as depicted in Figure 4 [52].

### Classical Models

Classical ML models rely on structured data and manually selected features for classification tasks. This category includes models such as logistic regression (LR), support vector machine, k-nearest neighbor, and naive Bayes. Among the included studies, 8 out of 11 (73%) applied classical models. Six (55%) of 11 studies implemented SVMs, which are commonly used for handling high-dimensional data and performing classification tasks [37-40,43,48]. In addition, 3 (27%) of 11 studies used naive Bayes models, 2 (18%) of 11 studies used KNN, 2 (18%) of 11 studies used LR, and 1 (9%) of 11 studies used decision trees [40,45,48].

Panagiotou et al [38] developed an SVM model with the potential application of point-of-care screening for cardiac fibrosis from VCG. They used 3 datasets, which cumulatively gave a dataset of 260 ECGs, of which 158 (60.8%) were from patients with CMR-confirmed fibrosis [38]. The data sources included the University Hospital Southampton (154 records, 108 with fibrosis), the PTB Diagnostic ECG Database (54 patients, 50 with fibrosis), and additional healthy controls from the PTB database (52 patients without fibrosis and CMR data) [53,54]. Preprocessing involved ECG transformation to VCG where applicable, ECG baseline removal, and wave boundary determination [55,56]. Feature selection initially included 9 features from all 3 planes of the VCG. They then reduced to the top 10 features using the SVMAttributeEval algorithm [57]. The best-performing SVM model achieved an accuracy of 82.36%, a sensitivity of 84.31%, and a specificity of 77.36%. Subsequently, Dima et al [37] refined the feature selection process using template-based, time-based, and statistical ECG features and spatial features from VCG, ultimately identifying 344 initial features which were narrowed to 25 key features for an SVM which achieved an accuracy of 82.1%, the sensitivity of 76%, and specificity of 87.5%, when tested across different databases.

Wieslander et al [47] assessed the ability of an LR model to improve the original manual Selvester scoring for individuals with left bundle branch block (LBBB). Data were amalgamated from 4 international institutions for individuals who had both ECG and LGE-CMR records. Across the 4 sites, they included 325 patients, 142 (43.7%) of whom had CMR-confirmed fibrosis. Data processing involved custom wave-processing software to identify waveform changes. They evaluated LR models for the detection, quantification, and localization of cardiac fibrosis by models that incorporated features from the manual LBBB Selvester score. Results showed that detection was improved in the LR to the manual score while quantification and localization were not improved. The LR had a sensitivity of 54%, a specificity of 84%, and an AUC of 0.72, while the manual score achieved an AUC of 0.60.

Melgarejo-Meseguer et al [39] trained an SVM for classifying the presence of fibrosis in individuals with hypertrophic cardiomyopathy (HCM) using 12-lead ECG data. The model was developed using a dataset of 43 ECGs selected by the research team, with 25 (58%) cases confirmed to have fibrosis by CMR. Data preprocessing included noise reduction with cubic splines, notch filters, QRS extraction, beat template creation, and grouping of beat templates into regional categories (lateral, anteroseptal, and inferior). Signal transformation was conducted using independent component analysis or principal-component analysis to isolate fibrotic signals. Feature extraction involved manually selecting statistical parameters of the QRS complex, such as power, SD, skewness, kurtosis, and number of local maxima, which were ranked to form a feature vector. The best-performing SVM, using principal-component analysis sorted by lowest SD, achieved an accuracy of 76.92%, a sensitivity of 75%, and a specificity of 80% for classifying fibrosis.

This work was then expanded upon in the study by Melgarejo-Meseguer et al [48] where 6 different linear and nonlinear classic and deep models were trained on 4 different databases. Of the 4 databases, 2 included simulated fQRS records, one database included 43 individuals with HCM and labeled fQRS, and one database included 300 ECGs of individuals with HCM who had CMR scar. The decision tree model achieved accuracies of 0.79 to 0.83 across the databases used. The SVM model achieved the highest performance for fQRS detection as an indirect measure of cardiac fibrosis, with 0.94 sensitivity, 0.88 specificity, 0.89 positive predictive value, 0.93 negative predictive value, and 0.91 accuracy. The naive Bayes model achieved the highest performance for direct fibrosis identification, with 0.47 sensitivity, 0.91 specificity, 0.82 predictive positive value, 0.66 negative predictive value, and 0.70 accuracy.

The included studies by Villa et al [43] and Goovaerts et al [40] are also closely connected, with Villa et al [43] validating and extending the original methods developed by Goovaerts et al [40] using external data. In the initial study, Goovaerts et al [40] developed an automated method for detecting and quantifying fQRS, an ECG marker associated with myocardial fibrosis. They used a dataset of 616 patients in normal sinus rhythm who had undergone ECGs before implantable cardioverter-defibrillator implantation. The presence of fQRS was labeled by 5 clinical observers based on predefined criteria, and cases with complete interobserver agreement were used as the ground truth for training the model. Data preprocessing involved noise reduction and voltage normalization, followed by segmentation of QRS complexes to accurately estimate fQRS and minimize misinterpretation from oscillations and noise in the signal, while also excluding irregular heartbeats. Features were independently extracted from each lead, using techniques like variational mode decomposition, phase-rectified signal averaging, and the count of peaks in the QRS complex. A set of 10 features was used to train an SVM, which achieved a sensitivity of 76% and a specificity of 92% for detecting fQRS, outperforming other models such as k-nearest neighbors, naive Bayes, and TreeBagger (as described in the Ensemble Models section).

Building on these findings, Villa et al [43] validated the method on a larger, multicenter dataset from 2 sources. The first dataset included 673 individuals before implantable cardioverter-defibrillator implantation, with 616 in sinus rhythm and 57 in atrial fibrillation. The second dataset comprised a retrospective set of 1259 ECGs from the European Comparative Effectiveness Research to Assess the Use of Primary ProphylacTic Implantable Cardioverter Defibrillators project, where fQRS was annotated independently by 2 clinicians. Villa et al [43] applied the same SVM-based approach to these datasets, achieving high specificity (92%) and a positive predictive value (86%) in the primary cohort, with robust performance in both sinus rhythm and atrial fibrillation settings. This validation of external data demonstrates the effectiveness and generalizability of the original method by Goovaerts et al [40] for identifying an indirect marker of fibrosis.

### Ensemble Models

Ensemble models combine multiple base models to function as a single, more robust model. By aggregating the predictions of various individual models, ensemble techniques like random forests (RFs) and gradient boosting aim to reduce overfitting and improve generalization to unseen data. These methods are particularly effective in handling high-dimensional data and capturing complex interactions within the dataset. Three (27.3%) studies reviewed applied ensemble models, of which 2 (18%) used RFs, 1 (9%) used gradient boosting, and 1 (9%) used TreeBagger [40,41,45].

Goovaerts et al [40] explored the use of an ensemble model, specifically TreeBagger, to detect fQRS in ECG signals. The study aimed to compare the performance of this ensemble model against classical ML approaches such as SVM, KNN, and naive Bayes (as described in the previous section). The TreeBagger model underperformed compared to other models in this study, achieving an AUC of 0.89, sensitivity of 64%, and specificity of 90% and it was less effective than SVM, which achieved an AUC of 0.95.

Khamzin [45] further explored the application of ensemble models by using both RF and Extreme Gradient Boosting (XGBoost) classifiers to detect myocardial scars based on simulated 12-lead ECG data. The study used a finite element model to simulate 20,000 ECGs from 10 patients with LBBB, half (50%) of which were simulated to have cardiac fibrosis. The data underwent principal-component analysis for dimensionality reduction, retaining components that explained up to 90% of the variance. The RF classifier achieved moderate performance with an AUC of 0.78, sensitivity of 47%, and specificity of 92%. In comparison, the XGBoost classifier outperformed the RF with an AUC of 0.83, sensitivity of 58%, and specificity of 95%. These results should be interpreted with caution as the study simulated high-dimensional and complex data based on a very small sample size.

Gemmell et al [41] developed an RF to detect and localize the presence, extent, and specific location of cardiac fibrosis within computationally generated models of the heart. The study focused on distinguishing fibrosis localized to either the left ventricle or the interventricular septum using features extracted from signal-transformed simulated ECG data. Initial model performance using an ensemble of 20 decision trees demonstrated promising results, with an accuracy of 76.6% for left ventricle localization and 83.33% for septal localization during 5-fold cross-validation. This accuracy improved significantly when the number of trees was increased to 1000, yielding an accuracy of 83.33% for left ventricle localization and 86.66% for septal localization. Leave one out cross-validation, also known as *k*-fold validation, a strategy in which each data point is used as a validation set once, produced an accuracy of 90.71% for left ventricle localization and 93.57% for septal localization. These findings highlight the capability of RFs to effectively leverage complex feature sets for precise cardiac fibrosis localization in computational models, suggesting its potential applicability in clinical settings where accurate fibrosis mapping is crucial.

### Deep Learning

Deep learning models, which use deep neural networks (DNN), offer advanced capabilities for analyzing complex temporal patterns and relationships within ECG data that traditional methods might miss. Convolutional neural networks (CNNs) are a subtype of DNNs designed for tasks involving spatial data. By leveraging deep learning models like CNNs, these models can extract nuanced features from high-dimensional datasets, leading to improved detection and classification of cardiac abnormalities. Of the included studies 4 out of 11 (36%) evaluated deep learning models [42,44,46,48].

Melgarejo-Meseguer et al [48] applied a multilayer perceptron (MLP) model, which is a type of feedforward neural network. As opposed to true deep models, which have many hidden layers, MLP has only one hidden layer; however, it is categorized here as a deep learning model for readability purposes. This model was used for ECG automated detection of both fQRS and cardiac fibrosis, achieving an accuracy of 0.78 across the databases used. In comparison to the classical models used in this study, the MLP model performed poorly, and therefore complete outcomes were not reported.

Gumpfer et al [42] evaluated a deep learning model for the automated detection of cardiac fibrosis using a dataset of ECGs and CMRs from 114 patients with known or suspected coronary artery disease. Included patients underwent both CMR and ECG to be eligible for the study. ECG data were preprocessed through cropping, scaling, and augmentation to compensate for the limited number of records. This augmented data, along with clinical data encoded into one-hot vectors, were used to train a CNN model architecture originally proposed by Strodthoff and Strodthoff [58] for identifying acute myocardial infarction. The model was further expanded with additional layers to produce a probability distribution for detecting cardiac fibrosis. The CNN model achieved a mean AUC of 0.81, sensitivity of 70%, specificity of 73%, and accuracy of 70.2% on a patient-level basis. When combined with clinical features, the performance of the model improved significantly, reaching a mean AUC of 0.89, a sensitivity of 70%, a specificity of 84.3%, and an accuracy of 78%. These results demonstrate the potential of CNNs, especially when combined with clinical data, for the accurate detection of cardiac fibrosis.

Tison et al [44] developed a deep learning model to identify patients with mitral valve prolapse (MVP) at risk for arrhythmias and myocardial fibrosis using ECG data. The study included 1349 patients with MVP, where ground truth was established using echocardiograms and CMR to confirm MVP and fibrosis. The CNN model was trained on preprocessed ECG data, including noise reduction and signal normalization, and achieved high performance with an AUC of 0.87 for detecting fibrosis.

Finally, Boribalburephan et al [46] investigated the use of image-based classification for detecting cardiac fibrosis and left ventricular ejection fraction below 50% using a dataset of 14,052 ECGs from 13,707 patients in Thailand, 27.11% of whom had cardiac fibrosis. The data, collected retrospectively, included 2 ECG formats: nongrid (old format) and grid (new format), with additional clinical features such as age, sex, smoking history, diabetes, hypertension, and dyslipidemia. Preprocessing involved converting PDF ECGs into images, removing grid lines when necessary, and cropping the images to maintain consistency across heartbeats. The study used 8 deep learning models, evaluating both single- and dual-task frameworks on the nongrid and grid data formats, and models combined with clinical features. The top-performing model for detecting cardiac fibrosis achieved an AUC of 0.84 for the old-format dataset and 0.81 for the new-format dataset. In comparison, cardiologists achieved lower AUCs of 0.63 and 0.66, respectively, highlighting the superior performance of the deep learning model.

The 11 studies represent promising initial strides toward the development of ML algorithms for clinical cardiac fibrosis detection from ECGs. Nevertheless, a thorough examination reveals substantial limitations within the current body of work that must be addressed in future studies.

## Discussion

### Principal Findings

In summary, our review identified 11 studies investigating the application of ML to ECG data for the detection of cardiac fibrosis. These studies used a variety of ML approaches, including classical models (8 studies), ensemble models (3 studies), and deep learning models (4 studies). SVMs were the most used classical model, while CNNs were prevalent among deep learning approaches. The best performance metrics varied widely across studies, with AUCs ranging from 0.72 to 0.97. The best-performing models achieved accuracies between 70% and 93% in predicting cardiac fibrosis. However, these results should be interpreted cautiously due to significant limitations in study designs, including small sample sizes, lack of diverse cardiac populations, and limited external validation. The reviewed studies demonstrate the potential of ML in detecting cardiac fibrosis from ECG data but also highlight the need for larger, more robust studies with diverse populations and rigorous external validation to establish the clinical utility of these approaches.

### Strengths in Comparison to Prior Work

The studies reviewed demonstrate several notable strengths in the application of ML to ECG data for cardiac fibrosis detection, particularly when compared to traditional manual methods. Researchers have explored a diverse range of ML techniques, including classical models like SVMs, ensemble methods such as RFs, and advanced deep learning approaches like CNNs, allowing for valuable comparisons between different methodologies. This diversity in approach and computational bolstering offers potential improvements over manual interpretation methods such as fQRS analysis and Selvester scoring. While fQRS meta-analyses reported a pooled sensitivity of 68.4% and specificity of 80.5%, some ML studies achieved impressive performance metrics, with AUCs reaching up to 0.94 and accuracies as high as 89%, suggesting superior diagnostic accuracy. Moreover, ML models address the subjectivity inherent in manual methods, which are susceptible to interobserver variability and diagnostic errors. Boribalburephan et al [46] found that their deep learning model outperformed cardiologists in ECG-based detection of cardiac fibrosis (AUC 0.84 vs 0.63-0.66). Once trained, ML models provide consistent and objective assessments, potentially reducing the risk of misinterpretation. The automated nature of ML analysis also offers a significant advantage in processing large volumes of ECG data efficiently, a crucial benefit given the time-consuming nature of manual interpretation by clinicians. Perhaps most importantly, ML models have demonstrated the ability to identify novel patterns in ECG data that may be difficult to discern through traditional human-derived methods, potentially uncovering new indicators of cardiac fibrosis. Several studies, such as that by Gumpfer et al [42], showed improved performance by combining ECG data with clinical features, highlighting the potential of ML to integrate multiple data sources for a more comprehensive analysis. This multimodal approach allows a more nuanced understanding of cardiac fibrosis, potentially leading to more accurate diagnoses and better patient outcomes. These strengths collectively underscore the promising potential of ML in ECG-based cardiac fibrosis detection, offering a path to overcome the limitations of traditional methods and providing a solid foundation for future research and clinical applications in this field.

### Limitations

#### Study Design Limitations

Of the reviewed studies, 5 had sample sizes of 42, 43, 80, 87, and 114, which are unlikely to be representative of the general population, and are technically problematic for ML. Recently, ML sample size criteria have been proposed, such as requiring a minimum of 10 samples per feature for classical models, an effect size ≥0.5, and an accuracy ≥80% [59,60]. The issue of sample size is also important as it concerns overfitting, a phenomenon where models trained on small sample sizes may become overly specialized to predict characteristics unique to that dataset. This issue is further compounded by the exclusion of critical biological variables such as sex, gender, age, and ethnicity, which are known to influence the presentation and progression of cardiac disease. The lack of consideration for these demographic factors can result in biased models that are overfit which will ultimately limit their clinical utility.

To address the challenge of small sample sizes in clinical settings, one potential approach is to apply transfer learning. This involves initially training a model on a large dataset and then fine-tuning it on a smaller one. This way, the model can learn fundamental properties of ECG signals that are transferable across different datasets, such as distinguishing between normal and abnormal ECG signals. Transfer learning is now feasible due to the accessibility of extensive public ECG datasets, such as the PTB-XL database [54]. We anticipate that future studies will increasingly adopt this methodology, thereby improving the reliability and generalizability of ML models in health care applications.

Consideration must also be given to the generalizability of both the study design and model development. With cardiac fibrosis exhibiting multifaceted characteristics across various conditions, broadening the spectrum of cardiology patients used for model training, rather than focusing solely on those with specific diagnoses, enhances generalizability. Of the reviewed studies, only Dima et al [37] and Panagiotou et al [38] included a broader cohort which included a variety of cardiac conditions. In addition, integrating dimensions of sex, age, and race into health care ML algorithms is essential for mitigating biases that could lead to diagnostic errors. This becomes particularly salient for conditions necessitating sex-, age-, and race-based risk stratification. While Wieslander et al [47], Melgareio-Meseguer et al [48], Gumpfer et al [42], Villa et al [43], and Tison et al [44] reported some patient demographics, it remains ambiguous how these demographics were included as input features in the development of the models. The absence of patient demographics integration, coupled with the exclusion of healthy controls in most studies, undermines the representativeness of the model to the broader population. The integration of other clinical information as well as a longitudinal analysis of change in each patient’s ECGs may further ML capacities for identification of fibrosis [60].

Data sources also contribute to inconsistency and lack of comparability across studies. Of the included studies, 2 studies incorporated VCG analysis [37,38,41]. Despite its lesser prevalence compared to ECG, VCG offers invaluable insights into the electrophysiological dynamics of the heart by providing a 3D representation of cardiac electrical activity derived from 3 leads, either directly acquired or mathematically extrapolated from the conventional 12-lead ECG [61]. Notably, the VCG has demonstrated heightened sensitivity in diagnosing specific pathologies, including atrial enlargement, right ventricular hypertrophy, and intraventricular conduction disorders, while affording superior spatial localization for informing and evaluating interventions [61]. Another technology, body surface potential mapping (BSPM), which involves using a greater number of electrodes across the thorax to provide higher-resolution electrophysiological representations, is being increasingly explored in AI research [62]. Nevertheless, the general clinical underutilization of VCG and BSPM persists in contrast to the widespread adoption of ECG, thereby constraining the interpretability and application of these technologies beyond specialist cohorts. However, future endeavors may harness VCG or BSPM input for computational models, potentially enriching diagnostic precision, and clinical insights.

Data undergoes preprocessing before input into ML models, a phase that entails using tailored techniques to optimize the data for improved learning effectiveness. This review of relevant studies revealed varying degrees of detail in describing preprocessing steps, with only some studies providing the requisite technical depth essential for reproducibility. Broadly, common preprocessing techniques encompass noise reduction, normalization, scaling, augmentation, transformation to VCG representations, and QRS segmentation. While the intricacies of these strategies surpass the technical scope of this review, details are available in the referenced studies.

Transparency in data processing is especially relevant when considering ECG artifacts. Sources of nonbiological noise in ECG signals, such as motion artifacts, electrode contact issues, and electrical interference, pose additional challenges. These artifacts can obscure the true cardiac signals, leading to inaccurate feature extraction and erroneous model predictions if not properly addressed during preprocessing. Oversight of these confounding variables risks creating models that appear to perform well in controlled settings but fail to deliver reliable results in real-world clinical environments. These confounding nonbiological signals may be more common in ECGs drawn from clinical environments due to a lesser degree of control over the circumstances where the reading was taken [63].

Accounting for all these factors is essential to ensure that models are being trained from relevant ECG features and to avoid study population-based biases. One possibility to address this is to adopt standardized preprocessing pipelines and reporting practices that could improve comparability across studies and facilitate external validation. Future research should prioritize the development of models that are not only accurate but also interpretable, ensuring that they can be reliably applied across various patient populations and clinical contexts. Various electrophysiological technologies, such as VCG or BSPM, may be considered either in conjunction with or as alternatives to ECG to furnish input data. A detailed account of data collection and processing methodology is needed for reproducibility, and the use of external validation using a dataset from a different institution can further improve model generalizability. Addressing these challenges will be crucial for the successful integration of ML-based ECG analysis into clinical practice, enabling more equitable and comprehensive methods for cardiac fibrosis detection.

#### ML Model Limitations

Exact model architectures were not consistently provided in the reviewed publications; however, the general limitations of each model type can be considered. Eight of the 11 studies used classical models [37-41,43,47,48], the most common being SVM, which was used in 6 of the studies [37-40,43,48]. Manual feature extraction is a major limitation in classical models, compared to the automatic feature extraction capabilities of deep learning. Manual feature extraction entails that models are trained to only evaluate features that are important to the human observer rather than intrinsic features that are mathematically more significant indicators of fibrosis. Furthermore, traditional SVMs may encounter computational limitations with high-dimension datasets, necessitate supplemental algorithms for handling time-series data, and may lack nuanced recognition of fibrosis characteristics. While DNN models are gaining prominence in ML for ECGs [51], we found only 4 DNN studies for cardiac fibrosis detection [40,43,46,52]. Mazomenos et al [56] published a conference abstract highlighting their use of deep learning for ECG-based detection of cardiac fibrosis in a cohort of 8813 patients to achieve an AUC of 80% and precision of 0.64; however, the brevity of the abstract prevented its inclusion as a primary study in this review. The use of deep learning methods, as demonstrated by Gumpfer et al [42], Tison et al [44], Wieslander et al [47], and Boribalburephan et al [46] offers several advantages for ECG data analysis, including hierarchical feature learning algorithms capable of distinguishing between simple (eg, waveforms) and intricate (eg, arrhythmias) patterns, automated feature extraction, adaptability to large and intricate datasets, adeptness in processing temporal data, and the ability to provide nuanced outputs, thereby enhancing clinical applicability.

To ensure the reliability of a model’s diagnostic capacity, it is essential to consider the validity of the ground truth, which constitutes the clinical basis for classifying cases into “fibrosis” versus “no fibrosis.” LGE-CMR is clinically considered the gold standard for identifying cardiac fibrosis; therefore, LGE-CMR should be the ground truth in model development using the ECG for fibrotic detection. Goovaerts et al [40], Villa et al [43], and Melgarejo-Meseguer et al [48] used fQRS as a proxy for cardiac fibrosis. Given the diagnostic limitations of fQRS, ML models that predict fibrosis based on fQRS approximations have limited diagnostic value, despite impressive outcome measures.

A further critical consideration in the assessment of ML models is the choice of model development and validation strategies. Ten of the 11 (91%) studies used cross-validation, a statistical technique in which the dataset is iteratively split into subsets for training, tuning, and evaluation. This leaves only a small subset of the original dataset for testing with a potentially skewed distribution of patient characteristics, which limits confidence in outcome measures. Further prioritization of validation using external data is necessary to improve the model’s generalizability and reduce the risk of overfitting. One of the 11 (9%) studies used bootstrapping. Bootstrapping is a technique that creates multiple training datasets by sampling with replacement from the original data, providing robust error estimates and CIs. However, it can be computationally expensive and may underestimate errors if the original dataset contains biases or is not representative of the true population distribution.

Embracing deep learning models coupled with large datasets that capture population diversity presents a promising avenue for addressing the limitations and enhancing the accuracy and clinical relevance of ML models for ECG fibrosis detection. Yet, there is a demand for further progress in harmonizing deep learning models with human judgment, as deep learning, unlike traditional ML models, is unable to fully elucidate the significance of extracted features in a manner that can be readily interpreted by humans [64]. However, methods that combine both deep learning and traditional ML classifiers are being increasingly applied to improve the interpretation of the models’ decisions. For example, the study by Tison et al [44] used a CNN to extract patient-level ECG features that were used as input to the interpretable gradient boosting model.

#### Limitations in Outcomes and Reporting

Across the 11 studies examined, a consistent pattern emerges: impressive outcome metrics are reported, such as high values for sensitivity, specificity, positive and negative predictive value, accuracy, and AUC. However, a closer examination reveals inherent challenges that hinder the clinical interpretation of these outcomes. Most of the studies, with the exception of those by Dima et al [37], Panagiotou et al [38], and Villa et al [43], solely present outcome metrics from internal cross-validation, without the use of external datasets. This restricts the scope of assessing the models’ generalizability and their relevance beyond the specific populations on which they were trained. A secondary limitation pertains to the incomplete reporting of outcome metrics which detracts from the holistic understanding of the models’ performance.

These limitations underscore a pervasive issue within the realm of health care ML research: the lack of consistency and transparency in outcome reporting. The Transparent Reporting of a Multivariable Prediction Model for Individual Prognosis or Diagnosis [65] guideline is being adapted to produce an AI-specific version [66] as recent assessments of health care ML studies revealed significant inconsistencies in reporting [67] and terminology [68]. As research in ML in ECG detection of cardiac fibrosis advances, reporting excellence must be achieved to fortify the scientific rigor and clinical utility of developed models.

#### Review Limitations

This scoping review has several limitations to consider. The relatively new and rapidly evolving nature of ML applications in ECG-based cardiac fibrosis detection means that despite thorough searches in multiple databases and outreach to authors, some relevant work may have been missed. The exclusion of conference abstracts without full papers, while necessary for ensuring methodological detail, may have omitted some early-stage research. The heterogeneity of the included studies, particularly in ML techniques, methods for establishing ground truth for cardiac fibrosis, and differences in methods documentation and outcome reporting posed challenges for direct statistical comparisons and meta-analysis. To accommodate a clinical audience, this review omits some technical details; however, methodological specifics of the ML approaches can be found in the original studies. In addition, the rapid pace of technological advancement in both ECG technology and ML algorithms means that earlier studies may not reflect the current state-of-the-art. Finally, reviewer bias can influence the selection, interpretation, and synthesis of studies, potentially introducing bias into the overall findings of a review.

### Considerations for Clinical Implementation

The current literature underscores both the promise and limitations of ECG-ML for detecting cardiac fibrosis. Although these studies represent pioneering efforts, their restricted sample sizes, absence of prospective trials, and limited diversity in patient demographics raise concerns about the generalizability and clinical utility of the findings. To translate this research into clinical practice, future studies should prioritize prospective randomized trials and the incorporation of broader demographic cohorts to ensure that ML models can serve a wide range of patients effectively. Furthermore, the use of more comprehensive datasets and external validation will enhance model robustness and reliability.

In the context of CMR resource limitations [69], ECG-based ML approaches present promising value. By guiding the allocation of CMR imaging resources or possibly circumventing the necessity for CMR, ECG-ML stands to enhance clinical efficiency and accessibility in the identification of cardiac fibrosis. When compared to manual methods, these emerging ML methods may reduce workload, identify subtle and novel patterns of fibrosis, and identify electrophysiologic-anatomical correlations between ECG and CMR. Furthermore, ML models may expand and expedite ECG-derived localization of fibrosis which is important in the prognostication of cardiomyopathy. A potential avenue for advancement lies in integrating fibrosis-detecting ECG models with other ECG diagnostic models to yield more comprehensive functional assessments. For instance, an integrated ECG model may identify a specific pattern of septal fibrosis as the cause of an observed conduction block.

To maximize the potential of ML models for ECG analysis, consideration must be made to their eventual implementation in clinical practice. There are numerous clinical trials and prospective evaluations of ML analysis of ECGs ongoing, but few reports of routine clinical implementation of these models [70,71]. Translational approaches to clinical implementation must address a wide range of challenges, including professional liability, systemic bias, surveillance and security, and integration within existing technologies and workflows [72]. The authors of a sepsis detection and management model, the first deep learning model to be implemented into routine clinical practice, outline steps to effective clinical implementation: workflow analysis, new workflow design, model and infrastructure development, integration and implementation, change management, and evaluation [73]. Incorporating ECG-ML into existing workflows requires careful consideration of both technical and clinical factors. Models must be able to integrate seamlessly with existing ECG infrastructure while enhancing clinician decision-making. Given the highly sensitive nature of cardiac fibrosis detection, clinicians must remain involved in interpreting ML outputs, especially during the initial phases of adoption. One promising approach is to combine ML-based fibrosis detection with other ECG diagnostic models to create more holistic assessments of cardiac function. For instance, integrating ML models with diagnostic tools for arrhythmias or conduction blocks can lead to more comprehensive evaluations of heart health, enabling more tailored treatment strategies.

Several barriers must be addressed before ECG-ML tools can be fully integrated into clinical workflows. One critical challenge is the need for high interpretability in ML models to ensure clinicians can understand and trust the outputs. In addition, practical concerns arise regarding the infrastructure required to support these models, including seamless integration with hospital electronic health record systems and ensuring robust data privacy and security. The computational costs associated with deep learning models remain significant, necessitating careful resource allocations. Systemic issues like professional liability and the mitigation of biases, especially regarding underrepresented patient groups, must also be addressed. Ethical considerations are crucial to prevent health care disparities from being exacerbated by biased data and model outputs. Finally, ensuring that these models comply with regulatory standards and can be smoothly integrated into clinical settings will require a multidisciplinary approach, combining technical advancements with policy reforms. Overcoming these challenges will enable ECG-ML models to enhance diagnostic accuracy while minimizing the risk of unequal treatment outcomes or increased clinician burden.

### Future Directions

To address these limitations, we propose further work to develop and train deep learning on large and diverse datasets to achieve efficient and accurate identification of ECG patterns indicative of cardiac fibrosis. While the current literature demonstrates promising strides in applying ECG-ML for detecting cardiac fibrosis, significant limitations must be addressed to advance clinical adoption. Future studies should prioritize larger, diverse cohorts, prospective randomized controlled trials, and standardized methodologies to improve generalizability and reproducibility. Standardizing data preprocessing and feature engineering, along with adherence to reporting guidelines such as the Transparent Reporting of a Multivariable Prediction Model for Individual Prognosis or Diagnosis, will enhance transparency and comparability. Incorporating external validation with independent datasets and integrating additional clinical data, such as longitudinal ECGs and advanced electrophysiological technologies like VCG or BSPM, can further refine these models. Collaboration between researchers and clinicians is crucial to ensure ML tools are developed with clinical relevance, enabling seamless integration into workflows and enhancing diagnostic accuracy. With the progression of research in this nascent domain, future qualitative and quantitative meta-analyses of models will be essential in facilitating deeper insights.

### Conclusions

This review underscores the potential of ML models applied to ECG data for detecting cardiac fibrosis, a key contributor to cardiovascular disease. Traditional methods like fQRS and Selvester scoring offer limited accuracy and require manual interpretation, whereas ML techniques show promise in enhancing diagnostic efficacy, accessibility, and precision. Despite these advancements, current research is hampered by small sample sizes, inconsistent methodologies, and a lack of external validation, limiting clinical applicability. Future studies should focus on larger, diverse cohorts, standardized data processing, and external validation to improve model robustness. The implementation of ML-based tools in clinical practice will require randomized controlled trials to demonstrate their efficacy and reliability in real-world settings, which is essential for widespread clinical adoption and improved patient outcomes. To enhance the applicability of ML-based ECG analysis, future research should prioritize external validation studies across diverse patient populations, ensuring that models are generalizable and clinically relevant. In addition, the exploration of underrepresented groups, including different races, ages, and comorbidities, will be crucial for developing inclusive and effective diagnostic tools. Addressing these gaps will accelerate the clinical potential of ML techniques for noninvasive cardiac fibrosis detection and improve resource allocation in health care. Ultimately, ML-based ECG analysis could expedite noninvasive cardiac fibrosis detection; however, more research is needed to fully realize its clinical potential and impact on patient care.

## Data Availability

All data generated or analyzed during this study are included in this published article and its supplementary information files.

## Appendix

Multimedia Appendix 1 PRISMA (Preferred Reporting Items for Systematic Reviews and Meta-Analyses) checklist.

Multimedia Appendix 2 Table of search strategies for all databases.

## Abbreviations

AUC: area under the receiver operating characteristic curve
BSPM: body surface potential mapping
CMR: cardiac magnetic resonance
CNN: convolutional neural network
ECG: electrocardiogram
fQRS: fragmented QRS
LGE: late gadolinium enhancement
LR: logistic regression
ML: machine learning
MLP: multilayer perceptron
MVP: mitral valve prolapse
RF: random forest
VCG: vectorcardiogram
XGBoost: Extreme Gradient Boosting
