# Supplementary material for: The Role of Machine Learning in the Detection of Cardiac Fibrosis in Electrocardiograms: Scoping Review
Source: JMIR Cardio. 2024 Dec 30;8:e60697. doi: 10.2196/60697 (PMC11730231; doi:10.2196/60697)
Supplement: Multimedia Appendix 2 [file cardio_v8i1e60697_app2.docx]

**The Role of Machine Learning in the Detection of Cardiac Fibrosis from Electrocardiogram: A Review**

Multimedia Appendix 1

| **Search** | **Search strategy** |
| --- | --- |
| PubMed | (("electrocardiogram" OR "ECG" OR "VCG" OR "Vectorcardiogram") AND  ("myocardial scar" OR "myocardial fibrosis" OR "cardiac scar" OR "cardiac fibrosis"  OR ("myocardial scar" AND ("fragmented QRS" OR "QRS fractionation" OR "fQRS" OR "selvester QRS" OR "selvester score"))  OR ("myocardial fibrosis"AND ("fragmented QRS" OR "QRS fractionation" OR "fQRS" OR "selvester QRS" OR "selvester score"))  OR ("cardiac scar" AND ("fragmented QRS" OR "QRS fractionation" OR "fQRS" OR "selvester QRS" OR "selvester score"))  OR ("Cardiac fibrosis" AND ("fragmented QRS" OR "QRS fractionation" OR "fQRS" OR "selvester QRS" OR "selvester score")))  AND ("artificial intelligence" OR "machine learning" OR "deep learning" OR "neural networks" OR "supervised learning" OR "unsupervised learning" OR "convolutional neural networks" OR "support vector machines" OR "generative" OR "random forests" OR "logistic" OR "model" OR "prediction" OR "classification" OR "modelling" OR "detection" OR "regression") |
| Scopus | TITLE-ABS-KEY ( "electrocardiogram" OR "ECG" OR "vcug" OR "Vectorcardiogram" ) AND ( TITLE-ABS-KEY ( "myocardial scar" OR "myocardial fibrosis" OR "cardiac scar" OR "cardiac fibrosis" ) OR ( TITLE-ABS-KEY ( "myocardial scar" ) AND TITLE-ABS-KEY ( "fragmented QRS" OR "QRS fractionation" OR "qrs" OR "sylvester qrs" OR "sylvester score" ) ) OR ( TITLE-ABS-KEY ( "myocardial fibrosis" ) AND TITLE-ABS-KEY ( "fragmented QRS" OR "QRS fractionation" OR "qrs" OR "sylvester qrs" OR "sylvester score" ) ) OR ( TITLE-ABS-KEY ( "cardiac scar" ) AND TITLE-ABS-KEY ( "fragmented QRS" OR "QRS fractionation" OR "qrs" OR "sylvester qrs" OR "sylvester score" ) ) OR ( TITLE-ABS-KEY ( "cardiac fibrosis" ) AND TITLE-ABS-KEY ( "fragmented QRS" OR "QRS fractionation" OR "qrs" OR "sylvester qrs" OR "sylvester score" ) ) ) AND TITLE-ABS-KEY ( "artificial intelligence" OR "machine learning" OR "deep learning" OR "neural networks" OR "supervised learning" OR "unsupervised learning" OR "convolution neural networks" OR "support vector machines" OR "generative" OR "random forests" OR "logistic" OR "model" OR "prediction" OR "classification" OR "modelling" OR "detection" OR "regression" ) |
| IEEE Explore | ("electrocardiogram" OR "ECG" OR "VCG" OR "Vectorcardiogram") AND ("myocardial scar" OR "myocardial fibrosis" OR "cardiac scar" OR "cardiac fibrosis" OR ("myocardial scar" AND ("fragmented QRS" OR "QRS fractionation" OR "fQRS" OR "selvester QRS" OR "selvester score")) OR ("myocardial fibrosis" AND ("fragmented QRS" OR "QRS fractionation" OR "fQRS" OR "selvester QRS" OR "selvester score")) OR ("cardiac scar" AND ("fragmented QRS" OR "QRS fractionation" OR "fQRS" OR "selvester QRS" OR "selvester score")) OR ("Cardiac fibrosis" AND ("fragmented QRS" OR "QRS fractionation" OR "fQRS" OR "selvester QRS" OR "selvester score"))) AND ("artificial intelligence" OR "machine learning" OR "deep learning" OR "neural networks" OR "supervised learning" OR "unsupervised learning" OR "convolutional neural networks" OR "support vector machines" OR "generative" OR "random forests" OR "logistic" OR "model" OR "prediction" OR "classification" OR "modelling" OR "detection" OR "regression") |
| Web of Science | "electrocardiogram" OR "ECG" OR "VCG" OR "Vectorcardiogram") (All Fields) AND ("myocardial scar" OR "myocardial fibrosis" OR "cardiac scar" OR "cardiac fibrosis" OR ("myocardial scar" AND ("fragmented QRS" OR "QRS fractionation" OR "fars" OR "selvester QRS" OR "selvester score")) OR ("myocardial fibrosis" AND ("fragmented QRS" OR "QRS fractionation" OR "fars" OR "selvester QRS" OR "selvester score")) OR ("cardiac scar" AND ("fragmented QRS" OR "QRS fractionation" OR "fars" OR "selvester QRS" OR "selvester score")) OR ("Cardiac fibrosis" AND ("fragmented QRS" OR "QRS fractionation" OR "fars" OR "selvester QRS" OR "selvester score"))) (All Fields) AND ("artificial intelligence" OR "machine learning" OR "deep learning" OR "neural networks" OR "supervised learning" OR "unsupervised learning" OR "convolutional neural networks" OR "support vector machines" OR "generative" OR "random forests" OR "logistic" OR "model" OR "prediction" OR "classification" OR "modelling" OR "detection" OR "regression") (All Fields) |
| DLBP | Myocardial scar \| Myocardial fibrosis \| Cardiac scar \| Cardiac fibrosis \| Fragmented QRS \| QRS Fractionation \| fQRS \| Selvester QRS \| Selvester Score |

Table 1. *Search strategies for all databases*
